# Older Adults’ Motivators and Barriers to Using Mindfulness Apps for Stress Management in Brain Health Interventions: Interview Study

**DOI:** 10.2196/79141

**Published:** 2026-05-22

**Authors:** Jasper W Scholl, Laura H H Winkens, Harm Veling

**Affiliations:** 1 Consumption and Healthy Lifestyles (CHL) Social Sciences Group (SSG) Wageningen University & Research Wageningen The Netherlands

**Keywords:** older adults, mindfulness apps, stress management, nighttime rumination, dementia risk reduction, digital multidomain lifestyle intervention

## Abstract

**Background:**

Population aging is driving a rapid rise in dementia cases worldwide, posing a major challenge for health care systems around the globe, including in the Netherlands. Digital multidomain lifestyle interventions, which target multiple lifestyle domains simultaneously, can protect against cognitive decline in at-risk older adults but struggle to sustain engagement. Addressing stress in these interventions is crucial, as it can directly increase dementia risk and may promote unhealthy behaviors in other domains targeted in these interventions, including physical activity, diet, and sleep.

**Objective:**

This study explores the motivators and barriers for Dutch adults aged 60 years and older to use mindfulness apps for stress management within digital multidomain lifestyle interventions. Despite their potential, it remains unclear which stress-related needs would motivate older adults to use mindfulness apps in these interventions and whether these apps effectively address those needs. Moreover, little is known about how older adults practice mindfulness independently in everyday settings alongside other lifestyle priorities.

**Methods:**

We conducted 15 semistructured interviews with former participants of a 26-week multidomain lifestyle intervention study (the “HELI” [Hersenfuncties na Leefstijlinterventie] study) that included a mindfulness app for stress management. Participants (8 females; age range 61-73 years) were first invited to describe their practices to improve brain health to see whether stress and mindfulness emerged spontaneously. Follow-up questions and scenarios explored experiences with stress and mindfulness before these concepts were explicitly introduced midway through the interview, providing insight into their roles in participants’ lives free from associations with the terms. Data were analyzed using template analysis.

**Results:**

Older adults reported experiencing fewer minor daily stressors than earlier in life and having developed effective coping strategies with age. These strategies often included elements associated with mindfulness, such as acceptance and deliberate attention to the present moment. However, many frequently worried at night about major concerns, including personal health, the well-being of loved ones, and global issues. These findings suggest that older adults may be more motivated to use mindfulness apps to cope with nighttime worry than with minor daily stressors. At the same time, older adults reported barriers to using mindfulness apps, including negative associations with the term “mindfulness” (eg, perceived as too spiritual) and challenges in maintaining focus during exercises.

**Conclusions:**

We discuss how older adults develop mindfulness skills with age and how these skills help older adults cope with daily stressors but not with nighttime rumination. Specifically, we argue that diminished cognitive resources at night, fewer distractions, and metacognitive patterns may sustain worry before sleep. This study highlights the importance of tailoring multidomain lifestyle interventions to the unique challenges of older adults. We also offer recommendations to present mindfulness in ways that help older adults stay focused during practice.

## Introduction

Dementia is a pressing societal concern. Worldwide, the number of people living with this disease is projected to almost triple, rising from 57 million in 2019 to 152 million by 2050 due to an aging population [1]. This carries significant socioeconomic implications, including in the Netherlands, where dementia is the leading cause of death and accounts for the largest share of health care expenditure, consuming 9.1% of the national health care budget [2]. If left unaddressed, the projected rise in dementia cases threatens to overwhelm health care systems across the globe. Dementia deeply impacts the lives of patients, their loved ones, and caregivers, and with no cure on the horizon, strategies to delay the onset of dementia are both urgent and worthwhile. Even a delay of 1 year in the onset of dementia could reduce its prevalence by 25% [3]. Digital multidomain lifestyle interventions may help achieve this delay but struggle to maintain user engagement [4]. This study explores the motivators and barriers to using mindfulness apps to address one of dementia’s key risk factors, stress, within these interventions.

Multidomain lifestyle interventions show promise in delaying dementia onset among older adults at elevated risk [5] by targeting modifiable risk factors linked to up to 45% of dementia cases [6]. Obesity, hypertension, and type 2 diabetes are key risk factors for Alzheimer disease, the most common form of dementia. These risks can be reduced through healthier choices in lifestyle domains such as physical and cognitive activity, diet, sleep, and stress management [7,8]. Multidomain lifestyle interventions target multiple lifestyle areas at once rather than focusing on a single domain, which may provide stronger protection against cognitive decline. Randomized controlled trials show that multidomain lifestyle interventions significantly enhance cognitive outcomes in at-risk older adults, while single-domain interventions often yield nonsignificant results [5]. The Finnish Geriatric Intervention Study to Prevent Cognitive Impairment and Disability, for example, assessed cognitive changes after a 2-year multidomain lifestyle intervention and found significant improvements in processing speed, executive function, complex memory tasks, and reduced risk of cognitive decline in older adults aged 60 to 77 years at risk of dementia [9].

The Finnish Geriatric Intervention Study to Prevent Cognitive Impairment and Disability trial suggests that targeting adults aged 60 years and older is worthwhile, and it may even be strategic to do so; unlike younger adults, older adults perceive dementia as a serious risk and face fewer time constraints, making them more receptive to preventive measures [10,11]. Digital interventions offer a promising way to reach older adults, as they are increasingly used by this group [12] and provide accessible, scalable, and cost-effective support [13]. A meta-analysis by Wesselman et al [4] found that digital multidomain interventions can significantly improve brain health outcomes, including global cognition, subjective cognition, and lifestyle risk scores. Unfortunately, sustaining long-term adherence remains a major challenge. The meta-analysis showed around 40% of participants dropping out in 6 digital multidomain intervention studies [4]. To impactfully reduce the societal burden of dementia, digital interventions must be designed to keep older adults engaged over time. Gaining insight into the motivators and barriers that influence engagement is crucial to improve the impact of these interventions.

This study aims to explore motivators and barriers that drive engagement with the stress domain within digital multidomain lifestyle interventions, given stress’s role as a key modifiable risk factor for dementia. Elevated stress can trigger excess cortisol release, increasing the risk of cognitive decline and dementia [14]. Stress can also indirectly increase the risk of dementia by leading individuals to unhealthy coping strategies such as drinking, smoking, poor diet, physical inactivity, and presleep rumination [15]. Stress occurs when a person perceives a situation as taxing, beyond their resources, and harmful to their well-being [16]. Stressors arise from past experiences, present challenges, or future threats, ranging from daily hassles (eg, traffic jams) to major traumas (eg, assault), life events (eg, losing a spouse), and ongoing struggles (eg, poverty) [16]. This study explores what drives or hinders older adults to use a tool for stress management that has gained traction in recent decades, mindfulness, within broader lifestyle interventions.

Mindfulness apps have emerged as well-researched and effective tools for reducing stress digitally [17]. Mindfulness is an umbrella term for practices, processes, and characteristics [18] that typically center around present-moment awareness, acceptance, and decentering [19]. Present-moment awareness involves deliberately focusing on moment-to-moment experiences, including body sensations, thoughts, and emotions. Acceptance entails adopting a nonjudgmental, curious stance toward one’s experiences rather than trying to control or change them. Decentering means perceiving thoughts and emotions as passing occurrences that do not always reflect reality and are separate from oneself. In mindfulness apps, these elements are trained through practices such as body scans and sitting meditation [20].

Meta-analyses show that mindfulness practice significantly reduces physiological stress markers like cortisol, blood pressure, and heart rate in various clinical and nonclinical populations [21] and that practice through apps can help reduce perceived stress [17,22] in both clinical and nonclinical populations. In 11 nondigital mindfulness programs reviewed by Hmwe et al [23], older adults reported benefits such as reduced stress and worry, improved sleep, sharper focus, and greater self-compassion. The mindfulness-to-meaning theory posits that mindfulness training achieves its benefits by accommodating a metacognitive state of awareness, where individuals observe perceptions, emotions, images, and thoughts without reacting to them [24]. This approach, known as the “third wave” of behavior therapy, differs from second-wave therapies like cognitive behavior therapy by encouraging individuals to observe their thoughts with psychological distance, rather than directly challenging the content of the thoughts [25].

To promote the benefits of mindfulness within multidomain lifestyle interventions, it is essential that older adults meaningfully engage with digitally delivered mindfulness exercises. Gaining in-depth qualitative insight into the motivators and barriers influencing this engagement is therefore imperative. A recent review by Hmwe et al [23] identified health benefits and positive reinforcement as key motivators and time commitment and difficulty maintaining focus as primary barriers to mindfulness practice among older adults. However, these findings are not directly applicable to the unique context of digital, multidomain interventions for brain health for 2 reasons. First, current studies have often focused on intensive, in-person programs led by professionals, offering limited insight into the challenges and motivations older adults may have when practicing mindfulness independently in digital formats. For instance, barriers such as time demands and distractibility noted by Hmwe et al [23] may be amplified when mindfulness is practiced in everyday settings alongside competing lifestyle priorities.

Second, while studies have explored the acceptability of mindfulness for addressing issues like breast cancer [26], chronic pain [27,28], and depression [10,29], it remains unclear whether stress and brain health are equally compelling reasons for older adults in the general population to engage in mindfulness. Specifically, it is unclear what stress-related needs might motivate older adults to use the stress management component of a broader lifestyle approach, or whether mindfulness effectively addresses those needs. Additionally, since there are indications that older adults may question the scientific basis of mindfulness for brain health [30], it is important to explore how they perceive its relevance and credibility in this context.

In addition, research is needed to understand how digital delivery influences older adults’ engagement with mindfulness exercises and how they perceive mindfulness as a tool for stress management and brain health. This study addresses this gap by exploring the following research question: “What are motivators and barriers for Dutch adults aged 60 years and older to use a mindfulness app in a multidomain lifestyle intervention for brain health?”

## Methods

### Study Design

In total, 15 semistructured interviews were conducted with former participants of the HELI (Hersenfuncties na Leefstijlinterventie; English translation: “Brain Functions after Lifestyle Intervention”) study [31] to identify motivators and barriers for using the mindfulness app in the study. The HELI and this study are both part of the “Maintaining Optimal Cognitive Function in Ageing” (MOCIA) [32] project. MOCIA aims (1) to signal an increased risk of cognitive decline and (2) to improve prevention in the Netherlands by developing a digital, multidomain lifestyle intervention. The HELI study supports MOCIA’s first goal by examining how a multidomain lifestyle intervention affects brain function. This study supports MOCIA’s second goal by evaluating whether mindfulness would be an acceptable addition to the project’s intervention.

The COREQ (Consolidated Criteria for Reporting Qualitative Research) [33] was used to report the study. We preregistered on the Open Science Framework (OSF) using the preregistration template of Haven et al [34] to make clear the research objectives and approach before starting the data collection. Supplementary materials can be found through OSF [35].

### Participants

Former participants of the HELI study were approached for an interview. The HELI study was a 26-week randomized, controlled, multidomain lifestyle intervention study in adults aged 60- to 75 years at risk for cognitive decline, conducted by the Radboud University of Nijmegen and Wageningen University [31]. The HELI study was divided into 4 separate waves: the first wave took place between August 2022 and February 2023, the second between January 2023 and July 2023, the third between June 2023 and December 2023, and the fourth between November 2023 and May 2024. This study included a total of 5 participants of wave 1, a total of 4 participants of wave 2, a total of 4 participants of wave 3, and a total of 2 participants of wave 4. This means that there was an interval of several months to 1 year between completion of the HELI study intervention and the interview for this study. Despite aiming for equal representation across waves in this study, the pool of eligible participants and willingness to participate varied between waves. Given the time interval between the HELI study and the interviews, asking participants to recall the intervention period would likely introduce recall bias. However, recalling the intervention period was not the focus of this study. Instead, we focused on participants’ current lifestyle behaviors and how they understand the origins of these behaviors. This approach provides insight into how lifestyle behaviors, particularly mindfulness practice, develop after completing a comprehensive lifestyle intervention. See the Materials section for the interview guide.

Participants of the HELI study were fluent in Dutch, lived within <50 km of Nijmegen or Wageningen in the Netherlands, and were at increased risk of cognitive decline. The risk of cognitive decline was operationalized as complying with 2 or more of the following risk factors: (1) BMI >30 kg/m^2^; (2) physical inactivity: <300 minutes of moderate-intensity aerobic physical activity or <150 minutes of vigorous-intensity aerobic physical activity per week, spread out over several days; (3) hypertension: systolic blood pressure >140 mm Hg and diastolic blood pressure >90 mm Hg; (4) hypercholesterolemia: total cholesterol >5 mmol/L or low-density lipoprotein cholesterol >3 mmol/L; (5) type 2 diabetes; and (6) cardiovascular disease. Exclusion criteria included technological illiteracy (complete incompetence in working with computers, apps, and online questionnaires), lack of internet access, cognitive impairment (as determined by Telephone Interview for Cognitive Status Modified), and medical conditions preventing safe participation (eg, epilepsy), not in line with the HELI study’s objective (eg, Alzheimer disease) or interfering with assessments (eg, ferromagnetic implants affecting magnetic resonance imaging). Participants were divided into 2 groups in the HELI study using block randomization (based on sex, age, education, and risk profile): a high-intensity group (n=52) and a low-intensity group (n=52). Both groups were encouraged to work on multiple lifestyle domains, including physical and cognitive activity, nutrition, sleep, and stress. Only the high-intensity group took part in the comprehensive lifestyle intervention.

Participants were approached for an interview for this study if they were in the high-intensity group, had expressed willingness to be contacted for future research, and had participated for at least 8 weeks. The HELI intervention focused on 1 lifestyle domain each week, and after 8 weeks, 2 of the 4 mindfulness-focused weeks had been held. All participants in the final sample of this study completed the intervention. We used stratified random sampling to ensure an equal sex distribution. This is important, as male individuals have been underrepresented in mindfulness research [36] and might be less supportive of mindfulness [37]. Participants were approached by email and asked to participate in an interview about their experiences with digital tools that they used during the HELI study. They could request additional information by phone or mail. Of the 36 individuals invited by email, 15 responded and participated in the interviews (female: n=8; highly educated: n=12; retired: n=10; ages 61-73 years). See Multimedia Appendix 1 for detailed demographics.

### Ethical Considerations

Ethics approval was obtained from the Wageningen University Research Ethics Committee (2023-060). Participants provided written informed consent prior to the interview. To protect participants’ privacy, personally identifiable information was removed from transcripts, and pseudonyms were used. Identifying information was stored securely in a protected location accessible only to the research team and kept separate from the anonymized transcripts, in accordance with university guidelines. Although participants were initially asked for optional consent to make anonymized transcripts publicly available, as documented in the preregistration, this plan was reconsidered after data collection due to the presence of sensitive information. Participants received a US $22 gift card as compensation.

### The Mindfulness App in the HELI Study

Participants had a combined intervention effort of 3 hours per week, of which 1.5 hours consisted of group sessions and 1.5 hours of home exercises. In the group meetings, participants received information and instructions about the exercises and shared experiences with other participants. The digital component of the intervention consisted of a platform (Ivido), where participants had access to (publicly available) apps with assignments. The VGZ mindfulness coach app [38] was used in the stress domain. Other digital components included a gamified walking app in the physical activity domain (Ommetje) [39], a module that offers strategies for dealing with cognitive decline in the cognitive activity domain (Keep your brain fit) [40], and a module for cognitive behavioral therapy for insomnia (CBT-I) in the sleep domain (i-Sleep) [41].

The VGZ mindfulness app is the central focus of this study. This app is developed by the Dutch insurance company VGZ and can be downloaded for free by all Android and iOS users. It offers a collection of 40 audio-based mindfulness exercises focused on breathing, attention, body scan, guided meditation, visualization, mantra, and yoga [38]. The exercises are sourced from established mindfulness programs by Jon Kabat-Zinn and Edel Maex. In the HELI study, a selection of 15 of the most popular exercises among users aged 60 years and older was made based on user data supplied by VGZ. Participants were expected to do 6 exercises per week with an average time investment of 15 minutes per session.

### Design

Semistructured interviews were conducted in this study, which is recommended for collecting rich descriptive data [42]. The semistructured interview provided structure to study our research question through a theoretical framework (ie, the Unified Theory of Acceptance and Use of Technology [UTAUT]-2 model) [43] and flexibility to explore and identify new themes.

Interviews were conducted until the data were sufficiently rich to generate meaningful themes that addressed our research aims. Consistent with our contextualist epistemology (Data Analysis section), we followed a notion of pragmatic saturation [44,45], as absolute saturation is neither attainable nor compatible with this perspective, and continued data collection may always yield new interpretations. Instead, pragmatic saturation involves a reasoned judgment that the data are sufficient to address the research questions. After 12 interviews, JWS and LW observed that several recurring patterns—such as older adults’ tendency to ruminate at night and to lose focus during mindfulness exercises—were sufficiently developed and meaningfully related to the research aims. Three additional interviews were conducted to further explore and refine these interpretations, after which data collection was stopped. We support transferability [46] by clearly describing the study context and participant group and by linking our interpretations to existing empirical and theoretical literature (Data Analysis and Discussion sections), enabling readers to judge their relevance to similar settings [44].

### Materials

We used a problem-centered interviewing approach [47] to design our interview guide. Here, the researcher enters the interview with certain priorities but considers the view of the respondent to determine what is relevant. For example, we start with a narrative opening account, asking participants to describe what matters most to them regarding brain health: “What are you currently doing to improve your brain health?” and “What else are you currently doing that might affect your brain health, good or bad?” By framing the conversation around brain health and deliberately avoiding terms like stress or mindfulness, we create space for participants to introduce these concepts on their own terms—if they view them as important. Whether stress and mindfulness are mentioned spontaneously, indirectly, or not at all provides insight into how participants perceive their relationship to brain health and how central—or peripheral—these concepts are in their everyday thinking and self-care practices.

Following the narrative opening account, we used detailing and ad hoc questions to reconstruct why respondents are engaging in those behaviors and not in others. We also introduced scenarios involving present-moment awareness and acceptance to see how these mindfulness concepts appear in daily life, without labeling them as such. The term “mindfulness” was only mentioned midway through the interview to avoid socially desirable answers. We used the UTAUT-2 model [43] to ensure that relevant components of technology acceptance and use are assessed, including performance expectancy, effort expectancy, social influence, facilitating conditions, hedonic motivation, and habit. For example, we ask directly about social influence: “Did you talk to people about the fact that you are or were doing mindfulness? How did they respond?” We also presented a simplified version to participants at the end of the interview to confirm which factors they found relevant. We chose UTAUT-2, as it most accurately predicts technology acceptance [48] and is tailored to a consumer context where other technology acceptance models (eg, UTAUT-1) are not [43].

The interview guide underwent multiple rounds of development. JWS made a first draft by drawing inspiration from other studies in the field [49] and the UTAUT-2 framework. JWS discussed this version with LW. JWS and LW opted to seek feedback on the interview guide from a group of colleagues, including a professor in qualitative methodology with a background in international development studies and 3 PhD candidates from similar fields. This group of researchers suggested the problem-centered approach. JWS adjusted the interview guide with this feedback, refined this version in a follow-up meeting with the full professor in qualitative methodology, and sent this version to LW and HV, who reviewed and approved the interview guide. Finally, we conducted a pilot interview with someone (male aged 63 years) who participated in an intensive lifestyle intervention that was not the HELI study and who had experience with mindfulness exercises. Based on this pilot interview, we refined the interview guide with minor adjustments, such as not explicitly mentioning the word “scenario” and shortening the introduction.

### Procedure

Interviews were scheduled by email, and participants were asked to read the attached information letter and provide written informed consent. The interviews took place at a location preferred by participants (ie, at home or at the university) between October 1, 2023, and January 8, 2024. In the interviews with participants 1, 3, 6, and 15, there was another person (ie, spouse or partner) present nearby, as the interviews took place in the living room. All interviews were conducted by JWS (from here on referred to as “the interviewer”). JWS has completed PhD courses for advanced qualitative research methods and has experience doing interviews. Participants were informed that the study centered around their experiences with digital tools, without specifically referring to mindfulness, to allow stress- and mindfulness-related concepts to emerge naturally during the interview. Moreover, participants were informed that the researchers of the interview study were not affiliated with the HELI study and were told that they were free to express their honest opinions and experiences. Audio of the interview was recorded with an audio-recording device, and participants were notified once the recording started. Prior to the interview, the interviewer and interviewee introduced themselves and shared information about their lives (eg, living situation, occupations, hobbies, and passions) to accommodate participants. Interviewees were asked whether they wanted additional information—such as the interviewer’s personal motivation for conducting the research—before the start of the interview. Then, the interview began using the interview guide as a starting point, with impromptu follow-up questions when necessary. At the end of the formal part of the interview, participants were informed about the reason for the special interest in mindfulness and were asked if there was anything worth mentioning that had not yet come up during the interview. The recording was stopped after this question, and participants were notified of this. Participants were then thanked for their time and received their gift card. Interviews took between 63 and 109 minutes (mean 81.9, SD 13.1 minutes).

The interviewer promptly recorded their experiences and reflective thoughts after each session. JWS transcribed the recordings verbatim. The artificial intelligence tool Trint was used to get initial versions of the transcripts, and JWS adjusted them manually to correct errors and to transcribe fragments that Trint was not able to process.

### Data Analysis

In line with recommendations by Braun and Clarke [50], we explicitly articulate the epistemological position guiding this study. We adopt a contextualist perspective [51], which holds that knowledge is shaped by participants’ understandings, researchers’ interpretations, shared cultural assumptions, and research community standards for evaluating interpretations [52]. This perspective allows researchers to identify meaningful patterns shaping participants’ accounts within specific contexts. Rather than seeking a single definitive truth, this perspective recognizes the coexistence of multiple interpretations and understands rigor as emerging from grounding these interpretations in context [51]. Accordingly, rigor was established through transparency about researcher assumptions, grounding interpretations in participants’ descriptions, and situating findings within empirical and theoretical literature. In line with this approach, we provided a positionality statement on OSF [35] and engaged in ongoing reflexive practice throughout data collection and analysis. For example, JWS critically examined whether his own experiences with worry shaped follow-up questioning or coding; these assumptions were interrogated through dialogue with the second coder, comparison with participants’ accounts, and engagement with relevant literature.

Data were analyzed using template analysis [53], a codebook approach to thematic analysis that involves developing a coding template with deductive and inductive codes [50,51,53]. Template analysis consists of six steps: (1) data familiarization, (2) preliminary coding of a subset of the data, (3) organizing themes into clusters, (4) drafting an initial coding template, (5) applying the template to new data and refining, and (6) finalizing the coding template and applying it to the full data set.

JWS (PhD candidate, male) and Yvon Laarakker (student assistant with an MSc in Health Sciences, female) began by reading the first 4 transcripts (step 1) and independently coding them (step 2) using a predefined template based on the UTAUT-2 model. They then discussed discrepancies and duplicates (ie, different code names for the same interpretation), whether any codes need to be removed, adjusted, or added, and how codes could be clustered into preliminary themes (steps 3 and 4). In line with our contextualist perspective, coding differences were treated as opportunities for reflexive discussion to explore alternative interpretations and develop a coherent, theoretically informed analytic framework rather than a single “correct” coding. For example, JWS initially emphasized deductive codes aligned with the UTAUT-2 model, whereas Yvon Laarakker prioritized inductive codes. After discussing the model’s relevance, JWS and Yvon Laarakker concluded that performance expectancy and effort expectancy seemed to be the most salient factors in participants’ accounts of mindfulness acceptability. To explore these in greater depth, JWS and Yvon Laarakker prioritized adding inductive codes to create detailed subcategories within these domains and decided to drop other factors of the UTAUT-2 model (ie, social influence, facilitating conditions, hedonic motivation, price value, and habit). With this revised coding template, they individually coded the rest of the interviews and updated codes. JWS and Yvon Laarakker reviewed the remaining transcripts together and engaged in discussion to refine a coherent codebook and collaboratively develop the themes (step 5). This codebook was applied to all data (step 6). The data were analyzed using Atlas.ti (version 24; Atlas.ti Scientific Software Development GmbH). Finally, to strengthen the study’s trustworthiness, we shared a summary of the findings with participants by email and asked them whether our interpretations were recognizable and meaningful, as well as whether they had alternative interpretations to offer. Participants indicated that the analysis resonated with their accounts and did not suggest additional interpretations.

## Results

### Overview

This study explored motivators and barriers for Dutch older adults to use a mindfulness app in a broader lifestyle intervention for brain health. The interviews showed that none of the respondents integrated mindfulness practice into their lives after the intervention. This stands in contrast to other apps included in the intervention. For instance, the gamified walking app Ommetje [39] inspired 6 of the 15 respondents (5, 9, 10, 12, 14, and 15) to maintain a daily walking routine for up to a year after the intervention. Similarly, the CBT-I website i-Sleep [41] helped 4 respondents (5, 8, 9, and 14) form better sleep habits. As the intervention did promote lifestyle change in these areas, it is noteworthy that the mindfulness app did not motivate older adults to adopt mindfulness practice in their daily lives.

We identified 3 themes reflecting motivators and barriers to using the mindfulness app. Theme 1 shows how different experiences of stress in older adults’ lives may motivate mindfulness app use (motivator). Theme 2 highlights perceptions of mindfulness that prevent engagement (barrier). Themes 1 and 2 relate to the UTAUT-2 factor performance expectancy. Theme 3 captures challenges in practicing mindfulness that hinder engagement (barrier). This theme relates to the UTAUT-2 factor effort expectancy. For further descriptions of the themes and subthemes, see Table 1. The following section elaborates on each theme with quotes, some of which are shortened for clarity.

**Table 1:** Themes and subthemes identified in data analysis.

Themes and subthemes			Essence of the subtheme		Participants
**Theme 1: motivator: older adults and stress**					
	1.1: Hassles	Older adults experience fewer minor stressors in this life phase and deal with them better.		All participants	
	1.2: Worries	Older adults worried and felt particularly ill-equipped to manage worry at night.		1, 2, 4, 5, 7, 8, 10, 11, 12, 13, 14	
	1.3: Mindfulness apps to manage stress	Older adults already felt mindful and had alternative strategies for managing daily stress.		2, 3, 4, 6, 7, 8, 9, 11, 12, 15	
**Theme 2: barrier: perceptions of mindfulness**					
	2.1: Negative associations	Mindfulness carries negative associations, like “spiritual” or “nonsense.”		1, 2, 3, 4, 5, 6, 10, 11, 12, 13, 14	
	2.2: Clarity of mindfulness	Older adults do not understand what mindfulness entails.		1, 2, 3, 4, 5, 12, 14	
**Theme 3: barrier: experiences with mindfulness apps**					
	3.1: Mindfulness exercises are difficult	Older adults got distracted, felt impatient, and were unsure if they were doing it right.		1, 2, 5, 6, 7, 8, 10, 11, 13, 14, 15	

### Theme 1: Motivator: Older Adults and Stress

The first theme explores how older adults experience and manage stress, which emerged in 2 forms during the interviews: hassles and worries. While daily hassles did not seem to motivate older adults to use the mindfulness app, worries appeared to be a potential driver for engagement.

#### Subtheme 1.1: Hassles

We first cover minor stressors of short duration, or “hassles,” such as being stuck in traffic, schedule conflicts, or general life maintenance [54]. All respondents reported that these daily stressors were less common than in earlier stages of their lives. Retirees, in particular, highlighted that their lives had become significantly calmer and less hectic since leaving the workforce, while those still employed mentioned an occasional need to unwind from their busy work schedules.

Getting married, raising children, and working is a hectic time (...), but eventually, you reach a point where you’re ready for it—I think. Ready to not have to do it all anymore. (...) I don’t get frustrated by long lines at the supermarket anymore; I have all the time in the world now.

Respondent 3, male, aged 68 years

That has to do with life phase. (...) If someone now asks me to go in front of me in the line at the supermarket, I’m like: “Sure, go ahead.” So what? I’ve got plenty of time. (...) I notice that one of my sons tries to fit so much in a single day. I used to be like that too. (...) When you’re young, you want to do a lot, but you also have to. Now, I don’t have to do anything anymore.

Respondent 8, female, aged 64 years

Both retirees and those still working noted that they had become more skilled at managing hassles over the years. When faced with annoyances, common approaches included taking on different perspectives or using phrases like “it is what it is” to accept the situation. To cope with feeling rushed, many found taking a walk and focusing on nature to be especially helpful. These strategies overlap with the mindfulness concepts acceptance and present-moment awareness (Textbox 1).

**Mindfulness-like coping strategies in older adults.**

“Every time I go for a walk, I feel myself calm down. Whether it’s the clouds, the river, or the green grass, it’s like a switch just flips, and all my work stress melts away. (...) Being outside, breathing and taking in nature ... while walking, I find myself on a different stream of thought” [Respondent 14, female, aged 61 years].

“(...) I really dislike the notion that you can always ‘manage’ life. (...) You have a problem and it needs to be solved. You feel annoyed and that feeling needs to disappear. You have a negative thought that you can’t bear and ... at some point, I noticed it negatively impact my life. I think many things are part of life and I’ve been trying to train myself to accept them. To just let it be” [Respondent 7, male, aged 73 years].

“There’s a saying I’ve remembered and still apply: ‘If something bothers you, change it if you can, accept it if you can’t, and learn to know the difference between the two.’ (...) for instance, if you hear a child crying on a plane, you can’t change it (...) empathize with the parents instead of blaming the child. (...) The sound does not go away, but it doesn’t bother you as much” [Respondent 2, female, aged 72 years].

#### Subtheme 1.2: Worries

Participants did report worrying about more significant stressors related to well-being. As shown in Table 1, a total of 11 of 15 participants reported worrying. Two main areas of concern surfaced: health and world issues. Health worries involved both their own well-being and that of loved ones and included both concerns about physical and cognitive health. Global issues, such as the wars in Ukraine and Gaza, along with growing polarization in the Netherlands, also troubled older adults—primarily because their grandchildren would grow up in this world (Textbox 2).

A strong theme was that worries came up specifically at night. During the day, participants managed their concerns by going for walks, engaging in hobbies, or talking to others. These activities kept their worries at bay and out of mind. However, at night, without distractions or the opportunity to talk things through, these worries surfaced and spiraled into rumination. Respondents also expressed frustration about nighttime rumination, as they felt it prevented them from falling asleep. They were worried that a lack of sleep would affect their (brain) health (Textbox 3).

**Health- and world-related worry topics reported by older adults.**

“Sometimes, I find myself brooding, and my thoughts just keep spinning. My daughter is pregnant and has health problems, which makes me worry about all the things that could go wrong. Panic really sets in during those moments. It’s all happening in my head. It’s really just nonsense because if something were to happen, I would handle it then, not now” [Respondent 2, female, aged 72 years].

“I usually skip over sections about the wars in Ukraine or Gaza in the newspaper because I don’t want to read about it anymore. (...) As I get older, I notice that what’s happening in the world really starts to weigh heavily on me, especially with grandchildren who have to live in it” [Respondent 11, female, aged 67 years].

“My daughter is going through a burnout and recently had EMDR, but she hasn’t shared much with me about it. I sometimes wonder if I should ask, but then I think it’s not really my place, and she’ll tell me when she’s ready. I end up having entire conversations with her in my head” [Respondent 14, female, aged 61 years].

**Older adults’ experiences of nighttime worry.**

“I’m a terrible sleeper and it really frustrates me. I wish things were different. When I lie awake at night in the dark, my thoughts become darker too. Sometimes I dwell on things that seem trivial during the day, but at night, I can’t stop the thoughts and my mind keeps spinning” [Respondent 10, female, aged 70 years].

“During the day, the thoughts don’t bother me as much. They bother me when I’m trying to fall asleep, so I try to fight them. (...) For instance, when someone is ill and I have no control over the situation. (...) During the day, I do things I enjoy or I can talk to people about it, but at night I keep ruminating (...) In those moments, I think to myself: ‘I need to fall asleep because it’s bad for my brain health if I don’t. Maybe I just shouldn’t stress so much about not sleeping’” [Respondent 5, female, aged 73 years].

“(...) During the day, when I go for a walk, I don’t ruminate—the thoughts just come and go. But at night, when I can’t do anything, my thoughts tend to spiral, and I get trapped in them. (...)” [Respondent 8, female, aged 64 years].

“I really don’t want to ruminate at night; that’s when my brain should get some rest. (...) I don’t mind thoughts about work, but they shouldn’t keep me from falling asleep” [Respondent 1, male, aged 71 years].

“When I go to bed, my thoughts start spinning. (...) It only happens then, when I’m alone and everything is silent. During the day, there are many distractions that keep those thoughts away” [Respondent 4, male, aged 72 years].

#### Subtheme 1.3: Mindfulness Apps to Manage Stress

Consistent with previous themes, daily stress did not motivate participants to use the mindfulness app; 10 of 15 felt it had no value at this stage of their lives. They explained, as outlined in subthemes 1.1 and 1.2, that they already practiced mindfulness effectively on their own and had developed strategies for managing hassles. It is important to note here that the HELI study positioned mindfulness as a tool for handling daily stress and not presleep worry (Textbox 4).

Overall, theme 1 shows that older adults experience less day-to-day stress but tend to worry about well-being, especially at night. Worry before sleep may serve as a stronger motivator for practicing mindfulness than daily hassles. Although we did not directly ask about presleep worry as a motivator—this theme emerged during data analysis—respondent 5 stated that if mindfulness could help her stop ruminating at night and fall asleep, she would start using the app “right away.”

**Perceived usefulness of mindfulness among older adults.**

“[When asked about mindfulness] ... I do that during my walks. It doesn’t need to be fancy. I often pause to look around, with both feet firmly on the ground, admiring a tree and pondering why its branches are shaped the way they are or why some might have broken off. (...) I started doing this when I was still working; I would walk off my troubles and trust them to nature” [Respondent 12, female, aged 72 years].

“When I came across mindfulness I thought, ‘Yoga works for me,’ and I kind of left it at that” [Respondent 7, female, aged 64 years].

“When I look back at my life, there were times when I would’ve been more open to using an app like this—(...) when I was trying to find inner peace and quiet my thoughts, years ago” [Respondent 15, male, aged 69 years].

### Theme 2: Barrier: Perceptions of Mindfulness

The second theme explores perceptions of mindfulness, showing two key points: (1) the term mindfulness carried negative associations and (2) its meaning is unclear.

#### Subtheme 2.1: Negative Associations

The term mindfulness and its presentation carried (strong) negative associations for 11 of 15 respondents, who referred to it as spiritual or nonsense. These associations emerged only after the term mindfulness was introduced midway through the interview. The concepts of present-moment awareness and acceptance, which we presented in scenarios before that point, did not elicit such associations.

It reminds me of those people who spend an hour discussing a painting ... or those who sip wine and say, “I think I detect a hint of blackberry.” (...) I sometimes tell my wife I find it ridiculous.

Respondent 3, male, aged 68 years

[While listening to the mindfulness app] ... I found it challenging when they said, “look for a place within yourself.” Now that I’m listening to it, I have to fight the urge to laugh out loud!

Respondent 14, female, aged 61 years

#### Subtheme 2.2: Clarity of Mindfulness

In total, 7 of 15 participants were also unsure what exactly falls under the umbrella of mindfulness. A question that was frequently asked was: “Is that mindfulness?”

It’s the term. [In a sarcastic, exaggerated tone] “Mindfulness!”. If you were to ask me what it means I’d say, “no idea.” My first association is meditation, but it’s much more than that.

Respondent 14, female, aged 61 years

Overall, in theme 2, we found that older adults had negative associations with the term mindfulness and its presentation and did not always fully understand what mindfulness entails, which may serve as a barrier for engagement.

### Theme 3: Barrier: Experiences With Mindfulness Apps

In the final theme, 11 of 15 participants found the mindfulness exercises challenging, creating an important barrier hindering use. Participants reported getting easily distracted during the exercises, experiencing impatience as they wanted to “get on with their lives,” and feeling unsure whether they were practicing it correctly. A common sentiment was that mindfulness “requires discipline that they did not have.” Participants cited several reasons for these challenges: the lengthy exercises and long pauses caused impatience and distraction, the passive “just listening” approach felt unengaging, and the instructions seemed vague or spiritual.

When they tell me to put my arms by my sides, that’s fine. But when they ask me to “sink into the mattress,” I’m like, “What the hell? (...) How am I supposed to do that? I can’t sink any further!”

Respondent 14, female, aged 61 years

With mindfulness, all you do is listen, so my mind just wanders. I feel like I didn’t do the exercises like they were meant to be done. I really tried, and I even have a book “meditating is something you have to learn,” but honestly, how on earth do people manage to meditate?

Respondent 5, female, aged 73 years

Difficulties practicing with the mindfulness app appeared to be a key barrier for respondent 5, who hoped it would help with nighttime rumination (see subtheme 1.3). While listening to one of the app’s exercises at the end of the interview, she said:

I really can’t keep this up for 15 minutes. My mind has already drifted off. The silent pauses just feel so long. I almost zoned out again.

## Discussion

### Principal Findings

This study examined motivators and barriers for Dutch older adults to use a mindfulness app to manage stress within a broader brain health intervention. A key finding is that none of the respondents embraced the mindfulness app as a meaningful addition to their lives, while other apps offered in the intervention, such as a gamified walking app, were successfully integrated into many of their daily routines. Thematic analysis identified 3 themes that explain the app’s limited reception. Theme 1 can be classified as a motivator, and themes 2 and 3 as barriers: (1) older adults lacked motivation for mindfulness apps, as they experienced fewer daily hassles and already had coping strategies to deal with daily stress; (2) older adults had negative perceptions of mindfulness (eg, that it is too spiritual) and did not fully understand what it is, hindering engagement; and (3) older adults struggled with mindfulness practice itself, with losing focus being the most common challenge.

### Comparison to Prior Work

An interesting finding related to the first theme was that older adults felt they had become better at managing stress with age through strategies akin to mindfulness, such as present-moment awareness and acceptance. This view aligns with research showing that mindfulness traits—present-moment awareness, acceptance, and decentering—all increase with age [55-59]. Mahlo and Windsor [57] explain these findings through the socioemotional selectivity theory [60], suggesting that mindfulness develops naturally as individuals become more aware of their finite time. This awareness encourages a stronger focus on enjoying the present and motivation for adopting an accepting attitude toward negative momentary experiences. They further propose that experiencing life changes in various domains (eg, social relationships, professional identities, and physical health) [61] deepens awareness of the fleeting nature of experiences. In support of this, research shows that older adults are generally less reactive to daily stressors [62], a pattern also observed in our study. Respondents reported that, with age, they had become better at managing everyday hassles by staying present in the moment, accepting situations as they are, and viewing their thoughts and feelings from a distance rather than becoming absorbed by them.

Older adults may have developed mindfulness qualities that help them cope with daily hassles, but the current findings suggest that older adults may still be inclined to rely on worry as a coping strategy for more significant issues. Older adults’ worries frequently revolve around important themes for well-being, including health and global political tensions [63-66]. Worry is a motivated response to manage these threats, rooted in the belief that it serves a protective function. Many individuals hold—whether conscious or not—positive metacognitive beliefs about worry, such as “worrying helps me cope” or “worrying keeps me prepared” [67,68], which leads them to engage with intrusive thoughts rather than letting them pass [69]. In some cases, worry can indeed be beneficial, serving as an emotional buffer or drawing attention to situations that require action, such as scheduling a medical check-up [70]. Given the potential impact of health and global issues on well-being, exploring threat scenarios and coping strategies through worry [68] may be more beneficial than dismissing these concerns too quickly. In contrast, hassles are often short-lived and less consequential, and stress or frustration serves little purpose in managing them. This aligns with the strength and vulnerability integration model, which posits that while stress management generally improves with age, uncontrollable and highly negative stressors remain challenging for older adults [71]. Over time, individuals may continue to view worry as useful for coping with such stressors but recognize that daily frustrations are unhelpful, leading them to adopt more mindful strategies for handling minor stressors [71].

Worry becomes problematic for brain health once it becomes excessive or interferes with sleep. In many cases, individuals can decenter and shift attention from worry once they feel that they have explored relevant outcomes [68]. However, those struggling with sleep may also negatively appraise and resist worry, with participants in this study reporting that worry “should not keep them from falling asleep” and describing trying to “fight” worry. These negative metacognitive beliefs can trigger a cycle of repetitive thinking [68,72]. This can cause individuals to shift between worrying about the trigger (eg, “What if my son gets in an accident?”) and the worry itself (eg, “I shouldn’t worry or I won’t sleep”), creating a sense of being “trapped in thoughts,” as one respondent put it. Sella et al [72] found that these negative metacognitions about worry contribute to sleep difficulties in “normally” aging older adults (ie, without insomnia), as those with such beliefs tend to rely on counterproductive strategies like thought suppression, rather than allowing thoughts to pass and shifting attention.

Cognitive fatigue and an absence of distractions also make it particularly challenging to manage worry in such a mindful way before sleep. Research suggests that older adults often have fewer cognitive resources at night, making it harder to block unwanted thoughts and leading them to rely on heuristics when processing concerns [73-77]. Even with the knowledge that it is better to allow thoughts to pass and not cling onto them, cognitive fatigue can make it more challenging to suppress dominant responses, such as rumination [78,79] and catastrophizing [80], at nighttime [77]. This may explain why participants reported dwelling on issues at night that seemed trivial during the day. Additionally, in line with observations by Charles [71] and Gould et al [81], older adults felt more vulnerable to their thoughts in the quiet of the evening without the distractions of the day.

In addition, metacognitive beliefs, cognitive fatigue, and the absence of distractions before sleep may help explain why older adults reported worrying specifically before going to sleep. The worry that may delay sleep reported by participants reflects a broader trend in the general population, with approximately 50% of older adults having difficulties falling or staying asleep [82]. Beyond the normal physiological changes in sleep patterns that accompany aging, dysfunctional metacognitions [83] and cognitive fatigue [78] can drive excessive worry that disrupts sleep and can harm brain health in older adults. Addressing these factors presents a promising opportunity in brain health interventions.

The mindfulness app offered in the multidomain intervention was rarely used for worry before sleep by our participants, despite its potential to counter unhelpful metacognitions through acceptance and psychological distancing. This may be because the HELI study presented the app primarily as a tool for managing daily stress, while sleep was addressed in a separate domain. This approach was reasonable, given that the sleep module (CBT-I) already included strategies for nighttime brooding, alongside support for a healthier circadian rhythm (eg, through sleep restriction). As a result, participants may not have seen its relevance for nighttime rumination. Since many already had strategies for coping with daily stress, they may have dismissed the app as irrelevant.

The analyses above suggest that it may be worthwhile to tailor mindfulness exercises better to the needs of older adults. A large cross-sectional study by Huberty et al [84] on users of the mindfulness app “Calm” (N=12,151) suggests that addressing presleep worry instead of everyday hassles is a promising possibility, as sleep difficulties were the most cited reason for use (72%), with older adults significantly overrepresented in this group. Other studies underscore that helping users recognize the value of mindfulness apps may equip them with the motivation to overcome other barriers, such as the unhelpful perceptions of mindfulness found in theme 2. In a study on a mindfulness-based positive aging intervention, Lomas et al [85] found that older adults faced remarkably similar barriers to practicing mindfulness, including associations with spirituality and a limited understanding, but were motivated to overcome these barriers, as they believed that its benefits outweighed any drawbacks. A review of qualitative studies by Hmwe et al [23] found that older adults practicing mindfulness for health issues like breast cancer, chronic low back pain, and depression cited perceived health benefits as the key reason for continued practice despite reporting similar obstacles. Some studies also market directly as “mindfulness studies,” possibly attracting participants who already believe in its benefits [38,86].

In addition, perceived usefulness (or “performance expectancy,” in terms of the UTAUT-2 framework) is the strongest predictor of mobile health adoption among older adults [12] and was likely a key factor limiting engagement in this study. This study underscores the importance of addressing the right needs with mindfulness and communicating benefits clearly within broader interventions, with worry before sleep as a compelling focus for older adults [83].

Finally, the barrier of finding mindfulness practice difficult in the third theme suggests that it may be worthwhile to present mindfulness concepts in more engaging ways. One respondent in this study was highly motivated to manage her worry with mindfulness but frequently became distracted during exercises, leaving her feeling as though she “wasn’t doing it right.” Participants noted several factors contributing to distraction, such as the length of the exercises, long silent pauses, unclear instructions, and an unengaging listening format. Lomas et al [85] suggested practical strategies to address most of these issues, including clear and structured guidance, concrete language, integration into existing routines, and keeping exercises under 10 minutes. We encourage practitioners to consider them when designing mindfulness apps. Even with these adjustments, individuals who worry before sleep may still find mindfulness meditation difficult. Banerjee et al [69] found that, especially when starting out with mindfulness practice, worriers can become so overwhelmed by their thoughts that they struggle to stay focused during listening exercises, leading them to view mindfulness apps as unhelpful [87]. Especially since mindfulness meditation often relies on mental imagery (eg, “imagine your thoughts as clouds”), it may be challenging for older adults with reduced cognitive resources at night to stay focused. While mindfulness exercises can help individuals manage worry once they are fully engaged, excessive worriers who are new to practice may benefit more from interventions that require more concrete and active participation, such as writing. Expressive writing [88], for example, encourages individuals to actively engage with worry rather than suppress it [89]. Thought records, commonly used in CBT-I, help worriers reframe unhelpful sleep beliefs through guided writing prompts, such as challenging the idea that 8 hours of sleep is essential [90]. Finally, writing interventions may be more effective when implemented earlier in the day, before cognitive resources decline [78]. Future studies can use these insights to design more engaging and effective tools for managing worry before sleep.

### Strengths and Limitations

The study has several strengths. First, we promoted the study as focused on brain health and only introduced the term mindfulness midway through the interviews. This allowed us to explore how stress naturally emerged in response to open-ended questions about lifestyle and understand the role of present-moment awareness and acceptance in older adults’ daily lives through scenario-based questions, free from associations with mindfulness. We found that older adults already use mindfulness-like coping strategies, and we identified worry as a promising target for brain health interventions. We also provide recommendations for addressing its mechanistic underpinnings. Second, we contribute to theoretical understanding by explaining why older adults may use mindfulness skills to manage hassles but not presleep worry, aligning with the strength and vulnerability integration model [71].

There are also limitations to consider. First, the sample may not represent the broader population of older adults at risk for dementia, as participants had completed a 6-month lifestyle intervention. Accordingly, we cannot fully rule out that some mindfulness-like coping strategies were influenced by exposure to the mindfulness app embedded in the intervention. Nevertheless, we consider our conclusions well-grounded, as many participants reported minimal engagement with the app, and the findings were interpreted in relation to existing empirical and theoretical literature on stress and aging. Second, our sample lacked diversity in socioeconomic position and cultural background. All participants were White and of Dutch origin, with 80% having a higher educational level, compared to 20%-30% in the general Dutch population [91]. To ensure that the needs of other demographics are taken into account in the development of brain health interventions, future research will require deliberate recruitment efforts. Unlike many mindfulness studies [36], our sample did achieve an equal sex distribution, providing insight into male perceptions of mindfulness.

### Conclusions

This interview study examines what motivates or prevents Dutch older adults from using a mindfulness app within a broader brain health intervention. Many respondents saw little value in mindfulness apps, making them less well-received than other intervention components. Though the app was positioned as a tool for general stress management, our findings suggest that it may have been more appealing if explicitly tailored to address presleep worry—an issue participants clearly recognized in their daily lives. We interpret these findings in light of the literature on aging and stress and offer recommendations for presenting mindfulness in ways that better align with older adults’ needs in multidomain lifestyle interventions.

The extent to which applying these insights improves brain health within the HELI study cannot yet be assessed, as the outcomes are not yet available. However, our findings suggest that the perceived relevance of intervention components in addressing specific issues of participants strongly determines engagement with them, which may influence their impact on brain health. Because stress is only one part of a broader lifestyle approach to preventing cognitive decline, other domains—including physical activity, nutrition, and sleep—must also align with older adults’ experiences and needs. Doing so is essential to maximize the effectiveness of comprehensive brain health interventions and to reduce up to 45% of dementia risk associated with modifiable lifestyle factors.

## Appendix

Multimedia Appendix 1 Demographic information and interview length.

## Abbreviations

CBT-I: cognitive behavioral therapy for insomnia
COREQ: Consolidated Criteria for Reporting Qualitative Research
HELI: Hersenfuncties na Leefstijlinterventie
MOCIA: Maintaining Optimal Cognitive Function in Ageing
OSF: Open Science Framework
UTAUT: Unified Theory of Acceptance and Use of Technology

## References

[1] GBD 2019 Dementia Forecasting Collaborators (2022). Estimation of the global prevalence of dementia in 2019 and forecasted prevalence in 2050: an analysis for the Global Burden of Disease Study 2019. Lancet Public Health.

[2] (2025). Feiten en cijfers over dementie. Alzheimer Nederland.

[3] Brookmeyer R, Johnson E, Ziegler-Graham K, Arrighi HM (2007). Forecasting the global burden of Alzheimer's disease. Alzheimers Dement.

[4] Wesselman LM, Hooghiemstra AM, Schoonmade LJ, de Wit MC, van der Flier WM, Sikkes SA (2019). Web-based multidomain lifestyle programs for brain health: comprehensive overview and meta-analysis. JMIR Ment Health.

[5] Kivipelto M, Mangialasche F, Ngandu T (2018). Lifestyle interventions to prevent cognitive impairment, dementia and Alzheimer disease. Nat Rev Neurol.

[6] Livingston G, Huntley J, Liu KY, Costafreda SG, Selbæk Geir, Alladi S, Ames D, Banerjee S, Burns A, Brayne C, Fox NC, Ferri CP, Gitlin LN, Howard R, Kales HC, Kivimäki M, Larson EB, Nakasujja N, Rockwood K, Samus Q, Shirai K, Singh-Manoux A, Schneider LS, Walsh S, Yao Y, Sommerlad A, Mukadam N (2024). Dementia prevention, intervention, and care: 2024 report of the Lancet standing Commission. Lancet.

[7] Livingston G, Sommerlad A, Orgeta V, Costafreda SG, Huntley J, Ames D, Ballard C, Banerjee S, Burns A, Cohen-Mansfield J, Cooper C, Fox N, Gitlin LN, Howard R, Kales HC, Larson EB, Ritchie K, Rockwood K, Sampson EL, Samus Q, Schneider LS, Selbæk Geir, Teri L, Mukadam N (2017). Dementia prevention, intervention, and care. Lancet.

[8] Moll van Charante EP, Richard E, Eurelings LS, van Dalen JW, Ligthart SA, van Bussel EF, Hoevenaar-Blom MP, Vermeulen M, van Gool WA (2016). Effectiveness of a 6-year multidomain vascular care intervention to prevent dementia (preDIVA): a cluster-randomised controlled trial. Lancet.

[9] Ngandu T, Lehtisalo J, Solomon A, Levälahti E, Ahtiluoto S, Antikainen R, Bäckman L, Hänninen T, Jula A, Laatikainen T, Lindström J, Mangialasche F, Paajanen T, Pajala S, Peltonen M, Rauramaa R, Stigsdotter-Neely A, Strandberg T, Tuomilehto J, Soininen H, Kivipelto M (2015). A 2 year multidomain intervention of diet, exercise, cognitive training, and vascular risk monitoring versus control to prevent cognitive decline in at-risk elderly people (FINGER): a randomised controlled trial. Lancet.

[10] Smith BJ, Ali S, Quach H (2015). The motivation and actions of Australians concerning brain health and dementia risk reduction. Health Promot J Austr.

[11] Vrijsen J, Matulessij TF, Joxhorst T, de Rooij SE, Smidt N (2021). Knowledge, health beliefs and attitudes towards dementia and dementia risk reduction among the Dutch general population: a cross-sectional study. BMC Public Health.

[12] van Elburg FRT, Klaver NS, Nieboer AP, Askari M (2022). Gender differences regarding intention to use mHealth applications in the Dutch elderly population: a cross-sectional study. BMC Geriatr.

[13] Domin A, Spruijt-Metz D, Theisen D, Ouzzahra Y, Vögele C (2021). Smartphone-based interventions for physical activity promotion: scoping review of the evidence over the last 10 years. JMIR Mhealth Uhealth.

[14] Ouanes S, Popp J (2019). High cortisol and the risk of dementia and Alzheimer's disease: a review of the literature. Front Aging Neurosci.

[15] Glanz K, Schwartz MD (2008). Stress, coping, and health behavior. Health Behavior and Health Education: Theory, Research, and Practice. 4th Edition.

[16] Lazarus RS, Folkman S (1984). Stress, Appraisal, and Coping.

[17] Linardon J, Cuijpers P, Carlbring P, Messer M, Fuller-Tyszkiewicz M (2019). The efficacy of app-supported smartphone interventions for mental health problems: a meta-analysis of randomized controlled trials. World Psychiatry.

[18] Van Dam NT, van Vugt MK, Vago DR, Schmalzl L, Saron CD, Olendzki A, Meissner T, Lazar SW, Kerr CE, Gorchov J, Fox KCR, Field BA, Britton WB, Brefczynski-Lewis JA, Meyer DE (2018). Mind the hype: a critical evaluation and prescriptive agenda for research on mindfulness and meditation. Perspect Psychol Sci.

[19] Tapper K (2022). Mindful eating: what we know so far. Nutr Bull.

[20] Crane RS, Brewer J, Feldman C, Kabat-Zinn J, Santorelli S, Williams JMG, Kuyken W (2017). What defines mindfulness-based programs? The warp and the weft. Psychol Med.

[21] Pascoe MC, Thompson DR, Jenkins ZM, Ski CF (2017). Mindfulness mediates the physiological markers of stress: systematic review and meta-analysis. J Psychiatr Res.

[22] Gál É, Ștefan S, Cristea IA (2021). The efficacy of mindfulness meditation apps in enhancing users' well-being and mental health related outcomes: a meta-analysis of randomized controlled trials. J Affect Disord.

[23] Hmwe NTT, Chan CM, Shayamalie TGN (2024). Older people's experiences of participation in mindfulness-based intervention programmes: a qualitative systematic review. Int J Ment Health Nurs.

[24] Garland EL, Farb NA, Goldin PR, Fredrickson BL (2015). The mindfulness-to-meaning theory: extensions, applications, and challenges at the attention–appraisal–emotion interface. Psychol Inq.

[25] Hayes SC, Follette VM, Linehan M (2004). Mindfulness and Acceptance: Expanding the Cognitive-Behavioral Tradition.

[26] Crane-Okada R, Kiger H, Anderson N, Carroll-Johnson R, Sugerman F, Shapiro S, Wyman-McGinty W (2012). Participant perceptions of a mindful movement program for older women with breast cancer: focus group results. Cancer Nurs.

[27] Luiggi-Hernandez J, Woo J, Hamm M, Greco C, Weiner D, Morone N (2018). Mindfulness for chronic low back pain: a qualitative analysis. Pain Med.

[28] Morone NE, Lynch CS, Greco CM, Tindle HA, Weiner DK (2008). "I felt like a new person." The effects of mindfulness meditation on older adults with chronic pain: qualitative narrative analysis of diary entries. J Pain.

[29] M Williams C, Meeten F, Whiting S (2018). 'I had a sort of epiphany!' An exploratory study of group mindfulness-based cognitive therapy for older people with depression. Aging Ment Health.

[30] Mace RA, Popok PJ, Hopkins SW, Fishbein NS, Vranceanu A (2023). Adaptation and virtual feasibility pilot of a mindfulness-based lifestyle program targeting modifiable dementia risk factors in older adults. Aging Ment Health.

[31] van Loenen MR, Remie LB, van Trijp MP, Jansen MG, Marques JP, Claassen JA, van de Rest O, Vermeiren Y, Smidt N, Sikkes SA, Deckers K, Zwan MD, van der Flier WM, Köhler S, Steegenga WT, Oosterman JM, Aarts E (2025). The effects of a multidomain lifestyle intervention on brain function and its relation with immunometabolic markers and intestinal health in older adults at risk of cognitive decline: study design and baseline characteristics of the HELI randomized controlled trial. JMIR Res Protoc.

[32] (2026). Mocia—Healthy Lifestyle, Healthy Brain. MOCIA.

[33] Tong A, Sainsbury P, Craig J (2007). Consolidated Criteria for Reporting Qualitative Research (COREQ): a 32-item checklist for interviews and focus groups. Int J Qual Health Care.

[34] Haven TL, Errington TM, Gleditsch KS, van Grootel L, Jacobs AM, Kern FG, Piñeiro R, Rosenblatt F, Mokkink LB (2020). Preregistering qualitative research: a Delphi study. Int J Qual Methods.

[35] Scholl J, Winkens L, Veling H (2024). Acceptance and use of app-based mindfulness by Dutch adults above the age of 60 in a multidomain lifestyle intervention aimed at improving brain health. Open Science Framework.

[36] Bodenlos JS, Strang K, Gray-Bauer R, Faherty A, Ashdown BK (2016). Male representation in randomized clinical trials of mindfulness-based therapies. Mindfulness.

[37] Katz D, Toner B (2013). A systematic review of gender differences in the effectiveness of mindfulness-based treatments for substance use disorders. Mindfulness.

[38] Van Emmerik AAP, Berings F, Lancee J (2017). Efficacy of a mindfulness-based mobile application: a randomized waiting-list controlled trial. Mindfulness.

[39] (2023). Ommetje-app. Hersenstichting.

[40] Reijnders JSAM, Geusgens CAV, Ponds RWHM, van Boxtel MPJ (2017). "Keep your brain fit!" Effectiveness of a psychoeducational intervention on cognitive functioning in healthy adults: a randomised controlled trial. Neuropsychol Rehabil.

[41] Van der Zweerde T, Lancee J, Slottje P, Bosmans JE, Van Someren EJW, van Straten A (2020). Nurse-guided internet-delivered cognitive behavioral therapy for insomnia in general practice: results from a pragmatic randomized clinical trial. Psychother Psychosom.

[42] Hill CE, Lambert MJ (2004). Methodological issues in studying psychotherapy processes and outcomes. Bergin and Garfield's Handbook of Psychotherapy and Behavior Change. 5th Edition [Nachdr.].

[43] Venkatesh V, Thong JYL, Xu X (2012). Consumer acceptance and use of information technology: extending the unified theory of acceptance and use of technology. MIS Q.

[44] Braun V, Clarke V (2019). To saturate or not to saturate? Questioning data saturation as a useful concept for thematic analysis and sample-size rationales. Qual Res Sport Exerc Health.

[45] Low J (2019). A pragmatic definition of the concept of theoretical saturation. Sociol Focus.

[46] Lincoln YS, Guba EG (1985). Naturalistic Inquiry.

[47] Witzel A, Reiter H (2012). The Problem-Centred Interview.

[48] Rondan-Cataluña FJ, Arenas-Gaitán J, Ramírez-Correa PE (2015). A comparison of the different versions of popular technology acceptance models: a non-linear perspective. Kybernetes.

[49] Parra DC, Wetherell JL, Van Zandt A, Brownson RC, Abhishek J, Lenze EJ (2019). A qualitative study of older adults' perspectives on initiating exercise and mindfulness practice. BMC Geriatr.

[50] Braun V, Clarke V (2023). Toward good practice in thematic analysis: avoiding common problems and be(com)ing a researcher. Int J Transgend Health.

[51] Madill A, Jordan A, Shirley C (2000). Objectivity and reliability in qualitative analysis: realist, contextualist and radical constructionist epistemologies. Br J Psychol.

[52] Pidgeon N, Henwood K (1997). Using grounded theory in psychological research. Doing Qualitative Analysis in Psychology.

[53] Brooks J, McCluskey S, Turley E, King N (2015). The utility of template analysis in qualitative psychology research. Qual Res Psychol.

[54] Proulx J, Aldwin C (2017). Stress and Coping Theory in Geropsychology.

[55] Feliu-Soler A, Soler J, Luciano JV, Cebolla A, Elices M, Demarzo M, García-Campayo J (2016). Psychometric properties of the Spanish version of the Nonattachment Scale (NAS) and its relationship with mindfulness, decentering, and mental health. Mindfulness.

[56] Lehto J, Uusitalo-Malmivaara L, Repo S (2015). Measuring mindfulness and well-being in Adults: the role of age and meditation experience. J Happiness Well-Being.

[57] Mahlo L, Windsor TD (2021). Older and more mindful? Age differences in mindfulness components and well-being. Aging Ment Health.

[58] Shallcross AJ, Ford BQ, Floerke VA, Mauss IB (2013). Getting better with age: the relationship between age, acceptance, and negative affect. J Pers Soc Psychol.

[59] Soler J, Cebolla A, Feliu-Soler A, Demarzo MMP, Pascual JC, Baños R, García-Campayo J (2014). Relationship between meditative practice and self-reported mindfulness: the MINDSENS composite index. PLoS One.

[60] Carstensen LL, Isaacowitz DM, Charles ST (1999). Taking time seriously. A theory of socioemotional selectivity. Am Psychol.

[61] Bernstein A, Hadash Y, Lichtash Y, Tanay G, Shepherd K, Fresco DM (2015). Decentering and related constructs: a critical review and metacognitive processes model. Perspect Psychol Sci.

[62] Neupert SD, Almeida DM, Charles ST (2007). Age differences in reactivity to daily stressors: the role of personal control. J Gerontol B Psychol Sci Soc Sci.

[63] Gonçalves DC, Byrne GJ (2013). Who worries most? Worry prevalence and patterns across the lifespan. Int J Geriatr Psychiatry.

[64] Hunt S, Wisocki P, Yanko J (2003). Worry and use of coping strategies among older and younger adults. J Anxiety Disord.

[65] Kogan JN, Edelstein BA (2004). Modification and psychometric examination of a self-report measure of fear in older adults. J Anxiety Disord.

[66] Wetherell JL, Le Roux H, Gatz M (2003). DSM-IV criteria for generalized anxiety disorder in older adults: distinguishing the worried from the well. Psychol Aging.

[67] Freeston MH, Rhéaume J, Letarte H, Dugas MJ, Ladouceur R (1994). Why do people worry?. Personal Individ Differ.

[68] Wells A (2010). Metacognitive theory and therapy for worry and generalized anxiety disorder: review and status. J Exp Psychopathol.

[69] Banerjee M, Cavanagh K, Strauss C (2017). Barriers to mindfulness: a path analytic model exploring the role of rumination and worry in predicting psychological and physical engagement in an online mindfulness-based intervention. Mindfulness.

[70] Sweeny K, Dooley MD (2017). The surprising upsides of worry. Soc Personal Psychol.

[71] Charles ST (2010). Strength and vulnerability integration: a model of emotional well-being across adulthood. Psychol Bull.

[72] Sella E, Cellini N, Miola L, Sarlo M, Borella E (2019). The influence of metacognitive beliefs on sleeping difficulties in older adults. Appl Psychol Health Well Being.

[73] Anderson JAE, Campbell KL, Amer T, Grady CL, Hasher L (2014). Timing is everything: age differences in the cognitive control network are modulated by time of day. Psychol Aging.

[74] Borella E, Ludwig C, Dirk J, de Ribaupierre A (2011). The influence of time of testing on interference, working memory, processing speed, and vocabulary: age differences in adulthood. Exp Aging Res.

[75] May CP, Hasher L (1998). Synchrony effects in inhibitory control over thought and action. J Exp Psychol Hum Percept Perform.

[76] Rabi R, Chow R, Paracha S, Hasher L, Gardner S, Anderson ND, Alain C (2022). The effects of aging and time of day on inhibitory control: an event-related potential study. Front Aging Neurosci.

[77] Yoon C, May CP, Hasher L (1998). Aging, circadian arousal patterns, and cognition. Cognition, Aging, and Self-Reports.

[78] Fawcett JM, Benoit RG, Gagnepain P, Salman A, Bartholdy S, Bradley C, Chan DK, Roche A, Brewin CR, Anderson MC (2015). The origins of repetitive thought in rumination: separating cognitive style from deficits in inhibitory control over memory. J Behav Ther Exp Psychiatry.

[79] Introzzi I, Andrés ML, Canet-Juric L, Stelzer F, Richard's MM (2016). The relationship between the rumination style and perceptual, cognitive, and behavioral inhibition. Psychol Neurosci.

[80] Galioto R, O'Leary KC, Thomas JG, Demos K, Lipton RB, Gunstad J, Pavlović JM, Roth J, Rathier L, Bond DS (2017). Lower inhibitory control interacts with greater pain catastrophizing to predict greater pain intensity in women with migraine and overweight/obesity. J Headache Pain.

[81] Gould CE, Gerolimatos LA, Edelstein BA (2015). Experimental examination of worry among older and young adults. Int Psychogeriatr.

[82] Ohayon MM (2002). Epidemiology of insomnia: what we know and what we still need to learn. Sleep Med Rev.

[83] Sella E, Carbone E, Borella E (2023). Do sleep-related metacognitive strategies shape my sleep? The relationships between strategies for controlling sleep-related intrusive thoughts and subjective and objective sleep quality in young adulthood and older age. Brain Sci.

[84] Huberty J, Vranceanu A, Carney C, Breus M, Gordon M, Puzia ME (2019). Characteristics and usage patterns among 12,151 paid subscribers of the Calm meditation app: cross-sectional survey. JMIR Mhealth Uhealth.

[85] Lomas T, Ivtzan I, Yong CY (2016). Mindful living in older age: a pilot study of a brief, community-based, positive aging intervention. Mindfulness.

[86] Mahlo L, Windsor TD (2021). Feasibility, acceptability, and preliminary efficacy of an app-based mindfulness-meditation program among older adults. Gerontologist.

[87] Crane C, Williams JMG (2010). Factors associated with attrition from mindfulness-based cognitive therapy in patients with a history of suicidal depression. Mindfulness (N Y).

[88] Pennebaker JW (2018). Expressive writing in psychological science. Perspect Psychol Sci.

[89] Wells A (2006). Detached mindfulness in cognitive therapy: a metacognitive analysis and ten techniques. J Ration-Emot Cogn-Behav Ther.

[90] McManus F, Van Doorn K, Yiend J (2012). Examining the effects of thought records and behavioral experiments in instigating belief change. J Behav Ther Exp Psychiatry.

[91] Van der Mooren F, de Vries R Steeds meer hoogopgeleiden in Nederland: wat voor beroep hebben ze?. Centraal Bureau voor de Statistiek (CBS).

