# Supplementary material for: Older Adults’ Motivators and Barriers to Using Mindfulness Apps for Stress Management in Brain Health Interventions: Interview Study
Source: JMIR Form Res. 2026 May 22;10:e79141. doi: 10.2196/79141 (PMC13241798; doi:10.2196/79141)
Supplement: Multimedia Appendix 1 [file formative_v10i1e79141_app1.docx]

## Table S1. Demographic information and interview length.

|  | **Interview length (in minutes)** | **Age** | **Sex** | **Education**** | **Retirement** |
| --- | --- | --- | --- | --- | --- |
|  |  |  |  |  |  |
| Participant 1 | 77 | 71 | Male | Higher | No |
| Participant 2 | 84 | 72 | Female | Higher | Yes |
| Participant 3 | 78 | 68 | Male | Higher | Yes |
| Participant 4 | 109 | 72 | Male | Lower | Yes |
| Participant 5 | 82 | 73 | Female | Higher | Yes |
| Participant 6 | 63 | 72 | Male | Lower | Yes |
| Participant 7 | 90 | 73 | Male | Higher | Yes |
| Participant 8 | 71 | 64 | Female | Higher | Yes |
| Participant 9 | 85 | 61 | Female | Higher | No |
| Participant 10 | >80^a^ | 70 | Female | Higher | Yes |
| Participant 11 | 64 | 67 | Female | Higher | Yes |
| Participant 12 | 91 | 72 | Female | Lower | Yes |
| Participant 13 | 95 | 62 | Male | Higher | No |
| Participant 14 | 84 | 61 | Female | Higher | No |
| Participant 15 | 73 | 69 | Male | Higher | No |

^a^ Audio recorder did not record a part of the interview as it had ran out of space on the SD-card. As a result, the exact interview length was not recorded.
